# The Graham Journal of Health and Longevity

**Published:** 1838

**Authors:** 


### Art. III.—The Graham Journal of Health and Longevity.

In the number for the 16th of April, of this zealous little periodical, the Divine, whose name has been made its cognomen, has responded to a couple of pages of ours, in the 42d No. of this Journal. It is, perhaps, generally known, through the United States, that "it is an object of the Graham Journal to put an end to the use of animal food." For expressing an opinion that this was neither desirable or philosophical, we have been charged with want of deliberation, want of candor, want of sense, want logic, want of physiological knowledge.

Mr. Graham, however, has not allowed his asperity to carry him so far as to charge us with eating meat. We should have thought, that an assailant of his system, who showed himself so thoroughly *impregnated* with *defects*, might have been safely left to his own destruction, but Mr. G. thinks otherwise.

He is amazed, at our referring to the appetite of the infant for milk, as evidence that animal food is proper for man; but if such diet were *improper* for its constitution, the author of that constitution would not have provided it. If animal food be necessary and healthful in infancy, at what subsequent period should it be abandoned? Why, undoubtedly, as in the case of the herbivorous animals, when the taste for it should be lost, or replaced by one for vegetable only. But who has ever witnessed such a change? The fact is, that the mother's milk is eminently vegeto-animal, and the taste for a mixed diet, manifested at the hour of birth, continues with us throughout life.

That taste does not, however, continue with the carnivorous and herbivorous, but after the period of lactation, it concentrates itself, in the former, on flesh, in the latter on plants. Man does not belong to either of these classes of exclusives, but is omnivorous, though, at the same time, capable of subsisting upon any one of the elements which nourish the animal kingdom. That he has a strong desire for flesh, is admitted even by the Graham school, as will appear from the following extract:

"Every mother, in every country where animal food is attainable," says the Doctor; "knows that children *prefer* animal food to vegetable," and we add that, every mother, in every country where coffee and tea are common beverages, knows that children, when they have been taught to love these beverages, prefer them to a glass of pure water. We acknowledge that where the mother makes a free use of flesh-meat it necessarily affects the idiosyncracy of the child, and affects the quality of her milk so as yet more strongly to predispose the child to form an appetite for flesh-meat; and when we add to these the odour of the animal food which almost continually surrounds the child, while it is cooking and while it is upon the table, and the fact that, such mothers almost universally begin at a very early period of infancy to give their children pieces of flesh to suck, and that, these children when they are old enough to be affected by the force or example constantly see all around them eating flesh, it is no marvel that they should almost universally learn to love it, and when they have learned to love it, it is almost a necessary consequence that the appetite, like the rum appetite and all others that are engrafted on the human system through a depravity of its natural instincts and sensibilities, should be stronger and more vehement and despotic than the natural appetite for natural and appropriate food and perhaps in most cases lead the child, when thus depraved, to *prefer* animal food to vegetable.

But we boldly affirm—and we fear not to stake our lives on the truth of the affirmation—that if any number of women be removed entirely from the use of animal food, and from the society of those that use it, so that they shall neither taste, smell nor see it, and subsist on a pure vegetable diet, and at the end of one year become mothers, and nurse their own children; and at a proper age accustom them to a diet of fruits and farinaceous vegetables plainly and simply prepared, every one of those children at the age of one year and upwards if there were millions of them, would instantly reject a piece of flesh-meat, with disgust and loathing, were it offered to them."

We have seldom met with a greater mass of error in the same compass. The mother eats flesh-meats until they impart their quality to her milk, and in that way the child acquires a taste for them—then, the savory odors of the kitchen regale its sense of smell, the delicious morceau given it to suck fascinates its taste—and still it has its natural propensity for animal food! still further, "all around" it gormandizes on the luxuries of the animal kingdom, and lead it astray by their example. But whence, *originally*, came the desire for this carnivorous indulgence? Whence these propensities of taste and smell? This adaptation of animal diet to them? If they do not belong, *naturally*, to our race, when and by what miraculous agency, were the habits here set forth established among us? If our preferences are artificial, are not those of the lower animals the same? But who taught the race of asses to browse on thistles instead of toads, or the latter animals to eat flies instead of grass? Why does the lamb scent out the thyme covered hills, and the young panther lie in wait for the fawn? Are all these *practices* traditional? It will be answered they are not, but the promptings of nature.—Then, why refer the preferences of man to another cause, or rather, to no cause at all? When we allow to the human race, natural desires for bread and flesh, taking them as types of the food derived from the vegetable and animal kingdoms, we travel with the common feeling, the common sense and the common praotice of the world. Example may modify, but can not create these desires.

We are told, "that every mother, in every country where tea and coffee are common beverages, knows that children, when they have been taught to love these beverages, prefer them to pure water." This is not true, when the object is to quench thirst. No discipline, example, or tradition, can make us prefer any thing to water when we are thirsty. But when we would enjoy the pleasures of taste or of constitutional excitement, we prefer tea and coffee and chocolate and wine and cider and beer and ardent spirits. Water will not gratify these desires, it will only satisfy our thirst, that is, our want of water. Now the love of sensation and excitement, is as much a part of human nature as the love of food and the love of water. It is gratified in a thousand different ways. Alcohol, opium, and tobacco are agents, that address themselves to this propensity, which, however, is not a yearning for alcohol, or opium, or tobacco, but for the excitement—the physical emotion—which they, and numerous other substances, raise in our systems. And thus man has not a natural appetite for this or that particular kind of meat, but for *animal food.* His preference for one variety over another, may be the result of the circumstances under which he has been placed; but the preference is not the propensity—it is but the choice which the propensity makes—the aspect under which the natural desire manifests itself. Men have an instinct for clothing—but the fashion of their clothes, and the intensity or extent of their indulgence in dress, will depend upon their means and the circumstances around them. This confounding of our natural propensities with their intemperate indulgence, and regarding the former as acquired, not original, is certainly a most superficial philosophy. Where did the Indians acquire that love of drunken excitement, which they manifest, from the commencement of the first paroxysm ?—Is it not innate? We cannot reason with the physiologist who should deny that it is. We do not, of course, speak of a natural relish for gin or whiskey, but for the excitement which they impart. The desire for this excitement is the basis of intemperance and the true remedy, is such a moral command over the propensity as will restrain and regulate its indulgence. While this is unachieved there is no safety.

The man who stimulates himself or eats meat to *excess*, violates the laws of his constitution and suffers. It is the business of philosphy and religion to keep us within proper bounds. If the Graham School would limit itself to an exposure of the bad effects of *excessive* indulgence in animal food, it would deserve commendation; but when it denounces *any* indulgence, and would persuade men that they have no *natural* taste for that kind of diet, they run into fanaticism, and many will run after them, because they are fanatical. Inscribe on your banner a bold absurdity, and you will be in no want of followers. The progress of Shakerism, not less than of Grahamism, is proof of this. The former is not more adverse to human nature than the latter. They are both commendable while seeking to prevent excessive or immoral indulgence ridiculous when they would *root out* the propensity.

